# Effects of a Violence Prevention Intervention Therapeutic Meeting With Aggression in Forensic Psychiatric Inpatient Care: Protocol for an Observational Study

**DOI:** 10.2196/74295

**Published:** 2025-10-09

**Authors:** Panagiotis Doinakis, Lilas Ali, Anna-Kari Sjödin, Peter Andiné, Eirini Alexiou, Sara Wallström

**Affiliations:** 1 Institute of Health and Care Sciences, Sahlgrenska Academy, University of Gothenburg, Arvid Wallgrens backe 1, Gothenburg, 413 46 Göteborg, Sweden, 46 0708911245 ext 0313437793; 2 Department of Forensic Psychiatry, Sahlgrenska University Hospital, Region Västra Götaland, Gothenburg, Sweden; 3 Centre for Ethics, Law and Mental Health (CELAM), University of Gothenburg, Gothenburg, Sweden; 4 Psychiatric Department, Sahlgrenska University Hospital, Region Västra Götaland, Gothenburg, Sweden; 5 Centre for Person-Centred Care (GPCC), Sahlgrenska Academy, University of Gothenburg, Gothenburg, Sweden; 6 Department of Forensic Psychiatry, Swedish National Board of Forensic Medicine, Gothenburg, Sweden

**Keywords:** mental health, forensic psychiatry, de-escalation, aggression, staff training, inpatient violence

## Abstract

**Background:**

Aggression and violence are prevalent in forensic psychiatric inpatient care. These behaviors significantly impact treatment outcomes, create challenging work environments for staff, and strain relationships between patients and caregivers. Managing such behaviors poses a formidable challenge that necessitates innovative approaches and evidence-based interventions. The Therapeutic Meeting with Aggression (TERMA) model is a staff training program designed to equip staff with strategies to de-escalate patient aggression, thus reducing violence and increasing patients’ and staffs’ perceived safety.

**Objective:**

The aim of this project is to evaluate the violence prevention model TERMA regarding perceived safety by patients and staff and adverse events within forensic psychiatric inpatient care. In addition, the project will investigate whether the organizational culture affects the implementation of the TERMA model.

**Methods:**

The project includes an observational study with a before and after design. Implementation of the TERMA model consists of an 8-seminar staff training program. Data sources include questionnaires, medical records, and registries. Quantitative data will be analyzed using descriptive and comparative statistics. To analyze changes between measurements, dependent sample 2-tailed *t* tests will be used for normally distributed data, and the Wilcoxon signed-rank test will be applied when normality is not met. The project will also include qualitative interview studies, which are planned to be analyzed using qualitative inductive content analysis.

**Results:**

Participant enrollment began in July 2023 and was concluded by the end of 2024. Data collection and analysis of quantitative data are expected to be completed by early 2026, after which the study findings will be submitted for publication in peer-reviewed scientific journals. Collection of qualitative data is scheduled for the second half of 2025 and 2026.

**Conclusions:**

This study can add valuable knowledge about the effects of the violence prevention model TERMA.

## Introduction

### Background

Aggression and violence are common within forensic psychiatric inpatient care, encompassing verbal and physical threats, as well as escalations that may result in physical injuries [1,2]. A meta-analysis found that 17% of patients in psychiatric inpatient care had been involved in at least one incident of interpersonal violence [3], and research indicates that the proportion is even higher, exceeding 20%, in forensic psychiatric inpatient care [4]. Studies from forensic psychiatric care report a higher prevalence of patients who are violent compared to acute care settings and general psychiatric hospitals [5]. Several factors could contribute to the escalation of aggressive incidences in forensic psychiatric inpatient care, including shortage of staff; insufficient knowledge or practice in aggression management; and changes in patient demographics, prevalence of mental health disorders, treatment approaches, and institutional policies [5].

In psychiatric (inpatient) care, aggression and violence have traditionally been seen as linked to mental illness and other individual patient characteristics. Therefore, response has centered on reactivity and control, and staff education has emphasized self-defense, control, and restraint techniques. However, over the last 2 decades, focus has shifted toward seeing aggressive patient behavior as a result of a complex interplay of diverse internal, external, and situational factors. Violent behavior may lead to injury but may also have other consequences, such as diminished therapeutic relationships [6], lower job satisfaction, and increased psychological stress [7,8]. Previous research on staff and patient experience of aggression and violence that resulted in coercive interventions (including seclusion, restraint, and medication) shows that incidents negatively affect treatment, work environment, job satisfaction, and therapeutic relationships between patients and staff [9]. Research also indicates that structured de-escalation models increase active listening between staff and patients that helped reduce seclusion and coercive methods within the ward [10]. In addition, the use of coercive measures as a response to violent behavior is problematic for both patients and staff and harmful for the therapeutic alliance. Moreover, patients experience these measures punitively [9].

To reduce the use of coercive measures, the recommended response to potential violence and aggression risk in forensic psychiatry is de-escalation of the situation [6]. It involves staff intervention before a situation reaches a point where more restrictive measures become necessary [6]. Several de-escalation models have been developed and are being used within psychiatric inpatient settings [11], including positive behavioral support [12,13], M4 (“managing the team, managing the environment, managing the patient, and managing aggression”) [14], the Bergen model [15], and the subsequent Therapeutic Meeting With Aggression (TERMA) model. A common objective among these models is the reduction of coercive interventions, but there are some distinct differences between the models [16]. Positive behavioral support focuses on bolstering staff confidence and modifying perceptions of challenging patient behavior with the primary objective of establishing a safe and secure environment [12]. It includes comprehensive staff training with a particular emphasis on enhancing communication skills and using tools to manage challenging patient behavior [12]. M4 centers on theoretical elements, including the analysis of organizational patterns of aggression, and places emphasis on risk management [14]. The Bergen and TERMA models place a central focus on treatment, communication, and the delivery of patient-centered health care within the specific context of forensic psychiatric settings. The models are designed to address and mitigate aggression and violence using a system of low-intensity interventions organized according to the patient’s aggression level, risk of violence, and history of previous violent acts [15]. At present, forensic psychiatric hospitals use a variety of de-escalation models, emphasizing both proactive and reactive measures to maintain a secure and therapeutic care environment. Communicative and cognitive de-escalation in the form of verbal discussion is one of the most common practices in de-escalation, emphasizing stimulus reduction and attempting to guide the patient away from unwanted behavior [17].

One obstacle to successful implementation of new work practices is that staff resist the change. A certain degree of resistance is common and expected when new practices are introduced [18,19]. Resistance to change refers to the reluctance or opposition displayed by staff when confronted with alterations in their work environment, practices, or procedures [20]. This resistance can manifest in various forms, such as skepticism, reluctance to adopt new practices, or active opposition to proposed changes [18,21]. Several factors contribute to resistance to change, including uncertainty and fear of the unknown, comfort with the status quo, lack of involvement in the change process, concerns about competence, previous negative experiences, workload and time constraints, and current cultural and organizational factors [22]. A number of factors are important to induce staff receptiveness to change, including work atmosphere, an effective framework of well-established procedures and methods, visible support for the change from leadership [18], transparent and open communication about the reasons for the change, the expected benefits, and how it aligns with broader organizational goals [22], and regular updates and opportunities for staff to ask questions or express concerns [18]. This significantly contributes to creating a sense of security and comfort, potentially resulting in a reluctance to embrace change or explore new techniques and work methodologies among experienced staff [21]. In addition, staff needs the necessary resources, tools, and technology to implement and sustain the changes effectively [18].

In Sweden, someone having been found guilty of a crime committed under the influence of a severe mental disorder can be sentenced to forensic psychiatric care instead of prison. Forensic psychiatric care is compulsory, and the sanction is not determined by time, as stipulated by the Swedish Courts’ Forensic Mental Health Act. Recent measurements indicate that the median care duration is 7.5 years but can be even longer [23]. Patients within forensic psychiatric care in Sweden represent a highly heterogeneous group [24]. Most common diagnoses include different forms of psychotic disorders, substance use disorders, and personality disorders. Comorbidity is prevalent, and 2 or 3 concurrent diagnoses are very common [25]. A large proportion of patients in forensic psychiatry are offenders who have committed violent crimes, and consequently, they present a distinctive set of challenges related to aggression and violence [24,26].

Delivering health care services within the framework of mandatory care poses challenges, including amotivation, noncompliance, and impediments to trust between patients and staff [27]. The indefinite duration of care adds complexity to the task of establishing a secure health care environment [23]. Forensic psychiatric inpatient care confronts these challenges and requires adaptation to ensure the effective implementation of treatment and the resulting discharge [28]. Violence and aggression are prevalent in forensic psychiatric inpatient care [3], posing a complex challenge that requires innovative and evidence-based interventions [4,16]. Current recommendations advocate for a proactive rather than a reactive approach to dominate staff education [29].

### Objectives

There is a lack of evidence regarding the effects of de-escalation models such as the TERMA model. Therefore, the aim of this project is to evaluate the violence prevention model TERMA regarding adverse events and perceived safety by patients and staff within forensic psychiatric inpatient care. In addition, the project will investigate whether the organizational culture influences the implementation of the TERMA model and the experiences of patients and staff in situations involving threats, violence, and the use of coercive measures. The project includes the following research questions:

- To what extent does the implementation of TERMA impact the perceived safety and perception of violence preventative culture in the wards of staff and patients in a forensic psychiatric inpatient setting?
- What is the effect of implementing TERMA on the frequency of incidents of aggression and violence, health care and occupational injuries, and the use of coercive measures in a forensic psychiatric inpatient setting?
- How does the organizational culture of a forensic psychiatric inpatient unit impact changes in perceived safety, incidents of aggression and violence, health care and occupational injuries, and the use of coercive measures?
- How do patients perceive threatening and violent situations, exposure to coercive measures, and the implementation of TERMA in a forensic psychiatric inpatient setting?
- What are the attitudes and perceptions of staff toward working with TERMA, how do they respond to threatening and violent situations, and what is the impact of these factors on the work environment in a forensic psychiatric inpatient setting?

## Methods

### Overview

This study uses an observational before and after design. The choice of TERMA was a pragmatic one, as it was being implemented in the clinic. However, the research design, including research questions, outcome measures, and methods of analysis, was not influenced by the clinic. The project was registered on ClinicalTrials.gov (NCT05932108) before starting the study. The protocol complies with the Standard Protocol Items: Recommendations for Interventional Trials (SPIRIT) [30,31], and the results will be reported according to established recommendations.

### Participants and Setting

Participants meeting the study’s inclusion criteria will be recruited from the Sahlgrenska University Hospital in Gothenburg. The forensic psychiatric clinic consists of 96 patient rooms, of which 12 are allocated to the high-security ward and 84 are dedicated to rehabilitation wards. The layout includes shared spaces such as a common room, a communal dining area, and a kitchen. Due to a shortage of health care rooms, some patients share rooms. In total, staff, comprising nurses, assistant nurses, psychiatrists, and other various professional occupations, amount to approximately 290 people. These various professional categories collaborate within health teams to formulate comprehensive health care plans and treatment strategies. The study encompasses both patients and staff within the forensic psychiatry setting. The inclusion and exclusion criteria for patients and staff are presented in Textbox 1.

Textbox 1. **Inclusion and exclusion criteria.**

**Inclusion criteria (patients)**

- Men and women aged ≥18 years committed to rehabilitation units
- Placed under the jurisdiction of the Mental Care Act (1991:1129) on Forensic Psychiatric Care
- Understand (speak and read) Swedish
- Assessed by the treating physician as mentally able to understand and consent to the study
- Willing to participate and sign a consent form

**Exclusion criteria (patients)**

- Minors (aged ≤17 y)
- Treated under legislation other than the jurisdiction of the Mental Care Act (1991:1129) on Forensic Psychiatric Care
- Cannot understand and speak Swedish and are thus unable to understand the study material
- Not assessed by the treating physician or assessed and deemed mentally unable to participate in research
- Unwilling to participate

**Inclusion criteria (staff)**

- Men and women aged ≥18 years working within forensic psychiatric inpatient care
- Understand (speak and read) Swedish
- Willing to participate and sign a consent form

**Exclusion criteria (staff)**

- Not directly involved in patient care within forensic psychiatric inpatient facilities
- Cannot understand and speak Swedish and are thus unable to understand the study material of the study
- Unwilling to participate

### Enrollment

All patients in the included wards will be assessed for eligibility by a member of the research team together with the ward staff and the responsible physician. Eligible patients will be given information about the study and asked to participate. Patients will be informed, both orally and in writing, that participation in the study is voluntary. They will also be assured that they can withdraw their consent at any time, and their decision to participate or not will not affect their care. Patients who indicate their willingness to participate will be issued a consent form. When returning the completed consent form, patients will receive the E13 questionnaire, which will include a randomly assigned identification number to ensure confidentiality throughout the research process.

All staff will be assessed for eligibility by a member of the research team. Eligible staff will receive detailed information about the study and be invited to participate. Information will be provided both orally and in writing. Staff who express their willingness to participate will be provided with a consent form. Upon returning the completed consent form, staff will receive 2 questionnaires (the E13 and the Organization Values Questionnaire [OVQ]), each coded with a randomly assigned identification number to ensure confidentiality throughout the research process.

### Design and Implementation of the TERMA Model

The TERMA model originates from Norway and has been used within both inpatient and outpatient psychiatric care. When it was first introduced in Sweden, it was given the name the Bergen model after the city it was developed in. At this time, it was adopted to align Swedish health care laws and adjusted to clarify a theoretical nursing approach. The Bergen model offers a specialized framework for managing aggression and violence within both inpatient and outpatient forensic psychiatric care [15]. After the evaluation of the Bergen model by Bergendahl et al [15], the model was adopted by another clinic in Sweden; further modifications were made; and the original name from Norway, TERMA, was reintroduced.

The core objective of the TERMA model is to proactively prevent aggression and violence by using a tiered system of low-intensity interventions, which are stratified based on the patient’s aggression level, risk of violence, and history of violent acts.

The TERMA model is rooted in the principles of compassionate health care and emphasizes treatment, communication, and effective management.

The foundation of TERMA acknowledges the inherent capabilities of all individuals, each possessing a unique self-awareness; distinct life experiences; expectations; needs; preferences; and available resources such as talents, interests, and education. Within TERMA, a central element involves a collaborative partnership between health care professionals and patients. They work together to formulate a personalized treatment plan, drawing upon the patient’s individual illness history and collectively identifying potential obstacles to effective care.

The TERMA model is structured in a “cascading” order, wherein the response to violence or aggression escalates if the interventions at one level do not prove enough.

These levels are designed to correspond with specific situational demands and environmental factors, optimizing their relevance and applicability. The *primary level* centers on the patient’s daily behavior and overall health status. It involves proactive measures aimed at understanding the patient’s emotional state, as well as managing stress and anxiety. Building trust and establishing balanced roles are key components at this stage. At the *secondary level*, the focus shifts to risk assessment and violence management. The aim is to cultivate mutual understanding between the patient and health care workers regarding the situation. The emphasis is on finding common ground and agreeing on acceptable solutions while preparing for further escalation if necessary.

When violence becomes inevitable, the *tertiary level* is activated. Here, the primary goal is to create a calm and secure environment for the patient, other patients, and staff. Clear leadership and low-intensity approaches are vital for diffusing tension, and physical restraint, if required, is executed with precision. Following the resolution of the situation, a situational analysis is offered to the patient to enhance understanding and prevent future incidents.

The implementation of TERMA is conducted through a staff training program consisting of 8 seminars. The seminars are divided into 4 theoretical sessions and 4 practical training sessions. The staff members first receive theoretical knowledge, followed by hands-on practice in restraint and self-defense techniques. This mixed format facilitates a holistic understanding of TERMA and equips staff with the skills necessary for implementation. The TERMA intervention will be uniformly implemented across all wards of forensic psychiatry at the Sahlgrenska University Hospital.

### Quantitative Sources and Collection of Data

Data will be collected from questionnaires, medical records, and administrative registers. Questionnaire data will be obtained from both patients and staff at baseline and 6- and 12-month follow-up. To assess perceived safety, data will be gathered from both patients and staff members using the E13 [15]. The E13 assesses self-reported safety, feelings of security, and violence preventative culture within the hospital wards. Respondents will be presented with 13 statements, and their level of agreement will be rated on a 4-point Likert scale, ranging from (1) “completely disagree” to (4) “completely agree,” with an additional “do not know” option available [15]. The questionnaire assigns a value of “1” to response options 3 and 4 for all items, except for the 3 negative items (items 4, 8, and 12), where a value of “1” is assigned to options 1 or 2 [15]. All other responses are scored as “0.” Thus, the sum score range for each questionnaire was 0‐13 [15].

The OVQ will be used to investigate the organizational culture in the wards. The survey will only be distributed to staff. The OVQ is based on the concepts of the competing value framework, which measures the 4 dimensions within each business: collaboration, creation, competition, and control [20]. These values compete in a very real sense for a business’s limited resources (funding, time, and people). The questionnaire contains 52 items, each rated on a 10-point Likert scale from “disagree” to “strongly agree” [20]. Data regarding incidents of threats and violence, patient and occupational injuries, and the application of coercive measures will be extracted from medical records and the local clinical registry known as MedControl PRO. These data points will be collected in 3 distinct phases: before the introduction of TERMA, at the 6-month juncture following implementation, and upon the culmination of a 12-month period. For staff members, supplementary descriptive data will be collected through a dedicated questionnaire, while background information pertaining to patients will be retrieved from their medical records.

### Outcomes

The primary outcome will be change in perceived safety reported by both patients and staff, as measured by the E13 scale total score (0‐13), between baseline and 6- and 12-month follow-up assessments. Secondary outcomes include changes in organizational culture, as measured by the OVQ and its subscales between baseline and 6- and 12-month follow-up assessments. Correlation between E13 and OVQ scores at baseline, 6-month follow-up, and 12-month follow-up will be analyzed. The number and severity of aggression incidents from 1 year before baseline to 1 year after conclusion of the study will be analyzed.

### Statistical Analysis

Descriptive statistics and comparative statistics will be used in the analysis. All data will be analyzed at a group level and reported using means and SDs. To analyze changes between the measurements, the plan is to use dependent sample 2-tailed *t* tests (if data are normally distributed) or the Wilcoxon signed-rank test (if data are not normally distributed). Regression models will be used to explore the relationship between organizational culture and the outcome of the implementation of TERMA. *P* values will be considered statistically significant if they are <.05.

### Qualitative Data Collection and Analysis

This project will also include qualitative design using semistructured individual interviews with both patients and staff members. Approximately 15 to 25 interviews are estimated to be suitable for answering the research questions in each study. The plan is to analyze data collected from the interview with qualitative inductive content analysis, but the decision if the method is appropriate will be based on the depth of the collected data and research questions [32-35]. Incorporating a qualitative design will facilitate greater understanding of patients’ perceptions of threatening and violent incidents, their experiences with coercive measures, and the use of TERMA within a forensic psychiatric inpatient environment. It will also offer valuable insight into staff perspectives regarding their approach to working with TERMA, their reactions to instances of threat and violence, and the resulting impact of these dynamics on the overall work environment within a forensic psychiatric inpatient setting.

### Patient and Public Involvement

To our knowledge, there was no patient and public involvement in the development or design of the TERMA model. In this research project, we sought to address this by actively involving patient representatives in various stages of this study. They were consulted about data collection and gave input on the choice of outcome measures to ensure that they are relevant from a patient perspective. In addition, we plan to engage them in the interpretation of the data. For the qualitative studies, the patient representatives will be engaged in the design of the interview guides as well as the interpretation of data. Finally, the patient representatives will be involved in the dissemination of the findings for all the studies.

### Ethical Considerations

The study has been approved by the Swedish Ethical Review Authority (2023-02451-01). All steps of the project have been designed to adhere to the principles outlined in the Declaration of Helsinki [36]. Written and verbal informed consent will be obtained from all participants, with clear information provided about their right to withdraw from the study at any time. The vulnerable nature of patients in forensic psychiatric care has been taken into consideration during the planning of the project, and special consideration will be taken when asking patients to participate in the project. They will also be reassured that participating in this research study will have no impact on their usual care. Permission obtained from the treating physician at each ward will be included in the project. Participants received no compensation to be part of the study. The methods used will be carefully considered to align with the study’s purpose, and the principles of confidentiality and integrity will be strictly maintained throughout the process. The collected data will be stored in a secure database maintained by the department and the university. This database is anonymized, and all analyses will be conducted on deidentified data, minimizing the risk of breaching confidentiality. The collected data are treated confidentially and presented in a way that ensures participants cannot be identified.

## Results 

Recruitment of participants began in July 2023 and concluded by the end of 2024. Collection of data from patient and staff questionnaires will be completed 13 months thereafter. Data from administrative registries will be collected retrospectively for the entire period. Data collection and analysis of quantitative data are expected to be completed by early 2026. Collection of data for the qualitative studies is planned for the second half of 2025 and 2026, and analysis is planned for 2026 and 2027. Results from this project will be published in peer-reviewed scientific journals and presented at international conferences.

## Discussion

This study protocol outlines an observational study aimed at evaluating the de-escalation method TERMA regarding adverse events and perceived safety by patients and staff within forensic psychiatry and if they are affected by organizational culture. Despite numerous de-escalation methodologies, there remains a significant gap in research regarding their use in forensic psychiatric care [11,16]. While the TERMA de-escalation model demonstrates promise, it currently lacks a robust body of research to support its application and assess its impact on perceived safety within forensic psychiatric inpatient wards. Previous evaluations of the Bergen model, which TERMA is closely related to, have shown positive influence on ward environment in psychiatric inpatient units [15]. The TERMA model prioritizes treatment, effective communication, and compassionate health care within forensic psychiatric settings, while also emphasizing improved communication and individualized action plans.

Various factors contribute to the current state of aggression within forensic psychiatry, with a prominent factor being the shortage of staff in forensic psychiatry [16,37]. In addition to assessing the intervention’s effectiveness, this project aims to identify if organizational culture may be a factor affecting the success of the implementation. Conducting research to comprehend this phenomenon and address the existing knowledge gap is crucial for multiple reasons, including ensuring patient care and safety, fostering a secure work environment for staff, enhancing education and practice, and holding relevance for the community [38]. Complex interventions, as described in this protocol, often involve multiple interacting components, making standardized methods and procedures less applicable [39]. This study combines questionnaires, data from medical records, and registries in the forensic psychiatric inpatient care setting, supplemented by qualitative data sources. A strength of the study is the use of validated questionnaires when assessing the effect of the implementation [39]. Data from these sources provide comprehensive insights into the impact of TERMA on perceived safety and perception of violence preventative culture. The shortage in research regarding de-escalation methods and their implementation in forensic psychiatry underscores the significance of this study. In addition, it will offer valuable information regarding the challenges and potential solutions when implementing a new de-escalation method within forensic psychiatry.

## Abbreviations

M4: managing the team, managing the environment, managing the patient, and managing aggression
OVQ: Organization Values Questionnaire
SPIRIT: Standard Protocol Items: Recommendations for Interventional Trials
TERMA: Therapeutic Meeting With Aggression
